# Butyrate Protects against Diet-Induced NASH and Liver Fibrosis and Suppresses Specific Non-Canonical TGF-β Signaling Pathways in Human Hepatic Stellate Cells

**DOI:** 10.3390/biomedicines9121954

**Published:** 2021-12-20

**Authors:** Eveline Gart, Wim van Duyvenvoorde, Karin Toet, Martien P. M. Caspers, Lars Verschuren, Mette Juul Nielsen, Diana Julie Leeming, Everton Souto Lima, Aswin Menke, Roeland Hanemaaijer, Jaap Keijer, Kanita Salic, Robert Kleemann, Martine C. Morrison

**Affiliations:** 1 Department of Metabolic Health Research, The Netherlands Organisation for Applied Scientific Research (TNO), 2333 CK Leiden, The Netherlands; wim.vanduyvenvoorde@tno.nl (W.v.D.); Karin.toet@tno.nl (K.T.); aswin.menke@tno.nl (A.M.); roeland.hanemaaijer@tno.nl (R.H.); ekzs@novonordisk.com (K.S.); robert.kleemann@tno.nl (R.K.); martine.morrison@tno.nl (M.C.M.); 2 Human and Animal Physiology, Wageningen University, 6708 WD Wageningen, The Netherlands; Jaap.keijer@wur.nl; 3 Department of Microbiology and Systems Biology, TNO, 3704 HE Zeist, The Netherlands; Martien.caspers@tno.nl (M.P.M.C.); lars.verschuren@tno.nl (L.V.); everton.soutolima@gmail.com (E.S.L.); 4 Nordic Bioscience, Biomarkers and Research, 2730 Herlev, Denmark; mju@nordicbio.com (M.J.N.); djl@nordicbio.com (D.J.L.)

**Keywords:** butyrate, obesity, non-alcoholic steatohepatitis, liver fibrosis, collagen, hepatic stellate cells, adipose tissue inflammation, gut

## Abstract

In obesity-associated non-alcoholic steatohepatitis (NASH), persistent hepatocellular damage and inflammation are key drivers of fibrosis, which is the main determinant of NASH-associated mortality. The short-chain fatty acid butyrate can exert metabolic improvements and anti-inflammatory activities in NASH. However, its effects on NASH-associated liver fibrosis remain unclear. Putative antifibrotic effects of butyrate were studied in Ldlr-/-.Leiden mice fed an obesogenic diet (HFD) containing 2.5% (*w*/*w*) butyrate for 38 weeks and compared with a HFD-control group. Antifibrotic mechanisms of butyrate were further investigated in TGF-β-stimulated primary human hepatic stellate cells (HSC). HFD-fed mice developed obesity, insulin resistance, increased plasma leptin levels, adipose tissue inflammation, gut permeability, dysbiosis, and NASH-associated fibrosis. Butyrate corrected hyperinsulinemia, lowered plasma leptin levels, and attenuated adipose tissue inflammation, without affecting gut permeability or microbiota composition. Butyrate lowered plasma ALT and CK-18M30 levels and attenuated hepatic steatosis and inflammation. Butyrate inhibited fibrosis development as demonstrated by decreased hepatic collagen content and Sirius-red-positive area. In TGF-β-stimulated HSC, butyrate dose-dependently reduced collagen deposition and decreased procollagen1α1 and PAI1 protein expression. Transcriptomic analysis and subsequent pathway and upstream regulator analysis revealed deactivation of specific non-canonical TGF-β signaling pathways Rho-like GTPases and PI3K/AKT and other important pro-fibrotic regulators (e.g., YAP/TAZ, MYC) by butyrate, providing a potential rationale for its antifibrotic effects. In conclusion, butyrate protects against obesity development, insulin resistance-associated NASH, and liver fibrosis. These antifibrotic effects are at least partly attributable to a direct effect of butyrate on collagen production in hepatic stellate cells, involving inhibition of non-canonical TGF-β signaling pathways.

## 1. Introduction

Liver fibrosis is a result of chronic liver damage for which non-alcoholic steatohepatitis (NASH) is one of the main causal factors [1,2]. Excessive food intake from energy-dense diets can induce hepatic lipids deposition (steatosis) which can progress to NASH (steatosis and inflammation) and fibrosis [3]. NASH-associated liver fibrosis is closely related to obesity, dyslipidemia, insulin resistance, white adipose tissue (WAT) inflammation, and gut dysfunction (increased gut permeability and changes in microbiota composition), all of which are drivers of disease progression [4,5]. To date, no approved treatment for liver fibrosis is available and little is known about how fibrosis development can be attenuated.

Liver fibrosis is characterized by excessive accumulation of extracellular matrix (ECM) proteins such as collagens [6]. Hepatic stellate cells (HSCs) are the main producers of these ECM proteins and therefore play a central role in liver fibrosis. During liver injury, transforming growth factor β (TGF-β) is the most potent fibrogenic cytokine and a key regulator in promoting HSC activation [7]. In response to TGF-β, the canonical signaling pathway (involving SMADs) and non-canonical signaling pathways (i.e., ERK, JNK/p38, Rho-like GTPases, PI3K/AKT) promote HSC activation [8]. The activation of these signaling pathways by TGF-β stimulates trans-differentiation of quiescent HSC to undergo a transcriptional and morphological program into proliferative HSCs that produce excessive amounts of ECM proteins.

Butyrate (BU) is a short-chain fatty acid that is produced by bacterial fermentation of dietary fibers in the colon and has been shown to have beneficial metabolic effects [9,10,11,12,13,14]. In humans, BU has been studied non-specifically and indirectly (by using probiotics and prebiotics to affect gut microbiota composition, which could potentially increase endogenous colonic butyrate production), which demonstrated that BU, in concert with other pre/pro-biotic induced changes, has satiety-increasing and body weight-reducing effects ([15] and references therein). These observations have been confirmed in rodents, where dietary supplementation with BU was shown to reduce food intake and attenuate body weight gain [16]. BU has also been shown to improve liver steatosis and inflammation [12,17,18]; however, it is unclear if these effects are secondary to its anti-obesogenic effects, or result from a direct effect of BU on these processes. Furthermore, while BU has been shown to improve many of the metabolic dysregulations that underlie development of inflammation and fibrosis in the liver, it is unknown whether BU can also reduce development of fibrosis in the context of obesity-associated NASH.

To study the potential anti-fibrotic effects of butyrate we used a well-established translational mouse model for diet-induced NASH and liver fibrosis: high-fat diet (HFD)-fed Ldlr-/-.Leiden mice. Under translational dietary conditions (i.e., using a HFD with a macronutrient composition and cholesterol content comparable to human diets) [17], these mice develop NASH with fibrosis in the context of obesity, insulin resistance, adipose tissue inflammation, and gut dysfunction, as is typical for many NASH patients [18]. In addition, these mice express underlying disease pathways relevant for human NASH, as demonstrated by recent transcriptomics, metabolomics, and proteomics studies [18,19]. To gain mechanistic insight into potential direct anti-fibrotic effects of BU, we performed in vitro studies with primary human HSCs.

## 2. Materials and Methods

### 2.1. Animal Experiment

The animal experiment was performed according to the standards of the Animal Care and Use Committee and ethically approved by an independent Animal Welfare Body (IVD TNO; approval number 3682/TNO-245). The ARRIVE guidelines were also adhered to. Ldlr-/-.Leiden male mice (94% C57BL/6J and 6% 129S1 genetic background) obtained from our breeding stock were group-housed (four to five mice per cage) in Macrolon cages in clean conventional animal rooms (relative humidity 50–60%, temperature ~21 °C, light cycle 07:00 to 19:00) in the American Association for Accreditation of Laboratory Animal Care (AAALAC)-accredited animal facility at TNO (Leiden, the Netherlands) with ad libitum access to food and water. Mice were matched into three groups of 15 mice per group based on body weight, plasma cholesterol and triglycerides, and blood glucose, which was the number of mice required to detect statistical differences in liver fibrosis. Two groups of mice (*n* = 15 each) were fed a high-fat diet (HFD; D12451, Research Diets Inc., New Brunswick, USA; containing 20 kcal% protein, 35 kcal% carbohydrate, 45 kcal% lard fat), or HFD+BU, in which HFD was supplemented with 2.5% sodium butyrate (303410 Sigma Aldrich, Steinheim, Germany). A reference group remained on standard laboratory chow (*n* = 15), a low-fat control diet (chow; Sniff-R/M-V1530, Uden, the Netherlands). The total treatment period was 38 weeks, during which the mice developed obesity- and insulin resistance-associated liver fibrosis [18,20], which was the main endpoint of this study.

Blood samples were collected via the tail vein from 5 hour-fasted mice, and body weight, food intake, and body composition measurements using echo-MRI were acquired at set intervals during the study. Gut permeability was analyzed after 37 weeks of dietary treatment, and after 38 weeks mice were sacrificed by gradual-fill CO_2_ asphyxiation after a 5 h fast. A terminal blood sample was collected via cardiac puncture, organs (liver and adipose tissue) were isolated and immediately snap-frozen in liquid N_2_ and/or fixed in formalin, and the mucosal microbiota was collected from the ileum and the colon as previously described [4].

### 2.2. Blood Chemistry

Analysis of cholesterol, triglycerides, insulin, alanine amine transferase (ALT), tissue inhibitor of metalloproteinases 1 (TIMP-1) in EDTA plasma, and whole blood glucose was performed as described previously [4]. LPS-binding protein (LBP) was measured in plasma with an ELISA kit following the manufacturer’s instructions (HK-205, Hycult Biotech, Uden, the Netherlands). The ECM-remodeling biomarkers PRO-C3 and PRO-C4, for type III and type IV collagen formation [21,22], and C4M and C6M to assess metalloproteinase-mediated degradation of type IV and type VI collagen, respectively [23,24], were measured in serum using competitive ELISAs (Nordic Bioscience, Herlev, Denmark).

### 2.3. Adipose Tissue Histopathology Analysis

Adipose tissue inflammation was assessed as described previously [17]. In short, the number of crown-like structures (CLS) were scored in hematoxylin-phloxine-saffron stained epidydimal WAT (eWAT) and mesenteric WAT (mWAT) cross sections in three non-overlapping fields (100 × magnification) per mouse. The number of adipocytes in the same fields were analyzed using Adiposoft [25] to express the number of CLS/1000 adipocytes.

### 2.4. Gut Permeability and Microbiota Composition Analysis

Gut permeability was assessed with an FD4 gut permeability assay, which assesses the ability of fluorescein isothiocyanate-labelled dextran (3–5 kDa FD4; Sigma, St. Louis, MO, USA) to leak through the intestinal lumen into the circulation, as described previously [4].

Microbiota DNA was isolated from mucosal samples of both the colon and ileum using the AGOWA Mag Mini kit (DNA Isolation Kit, AGOWA, Berlin, Germany), according to the manufacturer’s instructions. Metagenomic sequencing of the 16s rRNA gene spanning the v4 hypervariable region and data analysis was performed as reported previously [4].

### 2.5. Liver Histopathology and Biochemistry

Liver histopathology was scored using a standardized method for rodents that is based on the human NAS scoring system [26] in hematoxylin-Eosin-stained (HE) cross sections of the medial lobe. Hepatic fibrosis was analyzed in Sirius-red stained cross sections of the sinister lobe. Total steatosis, i.e., the sum of macrovesicular and microvesicular steatosis, hypertrophy (abnormally enlarged hepatocytes), and fibrosis for each mouse was determined as a percentage of total liver section affected. Hepatic inflammation was quantified by counting the number of inflammatory aggregates in five non-overlapping fields per mouse at 100× magnification (field of view 4.15 mm^2^) and expressed as the number of aggregates per mm^2^.

In addition, inflammatory chemokines were measured in liver biopsies homogenized in a 50 mmol/L Tris-HCL pH 7.4, 150 mmol/L NaCL, 5 mmol/L CaCl2, 1% Triton X-100 lysis buffer. Intrahepatic chemokines CCL3, CCL5, CXCL1, and CXCL10 were measured with a Quanterix chemokine panel according to the manufacturer’s protocol (Mouse 4-Plex Developer Kit; 100A-0497 Rev 01; Quanterix, MA, USA) on a SP-X Quanterix machine. Protein concentrations were measured with the BCA Protein Assay Kit (Thermo Fisher Scientific, Waltham, MA, USA) to determine chemokines per mg of protein.

Hepatic total collagen content was quantified based on hydroxyproline residues obtained from acid hydrolysis (QZBtiscol, Quickzyme, Leiden, the Netherlands) of the sinister lobe, and protein concentrations were measured in the same hydrolysates (QZBtotprot, Quickzyme) according to the manufacturer’s instructions.

### 2.6. Primary Human Hepatic Stellate Cell Experiments, Gene Expression Analysis, and Protein Expression Using Western Blot Analysis

Primary human hepatic stellate cells (HSC) (BioIVT, West Sussex, UK) were seeded on fibronectin-coated (Roche, Woerden, The Netherlands) 24-well culture plates and maintained overnight in stellate cell medium (STECM) supplemented with 2% (*v*/*v*) fetal bovine serum (FBS), 1% (*v*/*v*) antibiotic solution, and 1% (*v*/*v*) stellate cell growth supplement (all HSC seeding medium materials were from ScienCell, Carlsbad, CA, USA). HSC were incubated for 4 days in STECM supplemented with 1% (*v*/*v*) FBS and 1% (*v*/*v*) antibiotic solution with or without TGF-β1 (2 ng/mL; rh-TGFB1 R&D systems, Minneapolis, MN, USA), and TGF-β1 co-treated with BU (sodium butyrate, Sigma Aldrich; the same as was used in the animal experiment), acetate (sodium acetate, Sigma Aldrich), or caproate (sodium hexanoate, Sigma Aldrich).

Cytotoxicity was determined in triplicate per condition in conditioned medium using the CyQUANT LDH cytotoxicity assay (Invitrogen, Eugene, OR, USA), according to the manufacturer’s instructions. The cell-matrix fraction was hydrolyzed in 6M HCL and used for determination of collagen protein concentration based on hydroxyproline residues (QZBtiscol, Quickzyme) and total cell protein concentrations (QZBtotprot, Quickzyme), following the manufacturer’s instructions. Total protein levels were used to correct the collagen concentration per sample. Human procollagen 1α1 and plasminogen activator inhibitor 1 (PAI1) protein expression (R&D Systems, Abingdon, UK) were measured in conditioned medium collected after 24 h of stimulation.

In addition, next-generation sequencing was performed in RNA samples extracted from *n* = 6 unstimulated; *n* = 8 TGF-β; *n* = 8 TGF-β+BU conditioned HSC. RNA was isolated using the RNAqeous Microkit (Thermo Fisher Scientific) and RNA concentrations were determined spectrophotometrically, as described in [17]. The NEBNext Ultra II Directional RNA Library Prep Kit for Illumina (New England BioLabs, Ipswich, MA, USA) was used to process the samples, following the manufacturer’s instructions. Strand-specific messenger RNA sequencing libraries were clustered, and sequenced on a NovaSeq6000 system (paired-end 20M reads/sample, Illumina, San Diego, CA, USA) at Genomescan (Leiden, The Netherlands). The quality was checked with the Illumina data analysis pipeline RTA3.4.4 and Bcl2fastq v2.20, and filtered and trimmed using TRIMMOMATIC software. These trimmed files were merged and aligned to the reference genome (Homo_sapiens.GRCh38.gencode.v29) using the STAR 2.5 algorithm with default settings. Next, Htseq-count 0.6.1p1 was used to count the read mapping frequency per gene, resulting in count files (GSE179395) which served as input for the differentially expressed genes (DEGs) analysis using the Deseq2-method [27]. DEGs (*p* < 0.0001 and Z-score > 1.3) were used as an input for pathway analysis through Ingenuity Pathway Analysis (IPA) [28]. Based on gene expression of all known downstream target genes, IPA predicted activation or deactivation of an upstream regulator, as reported in [17].

Protein expression of cMyc was analyzed using western blot analysis. HSC from each condition (two pools of three wells per condition) were collected by scraping in ice cold lysis buffer [29], and protein content was determined (BCA Protein Assay Kit, Thermo Fisher Scientific). Protein samples (10 μg) were prepared, separated, and blotted, as previously described in [29]. The blotting membranes were treated with block buffer for 1 h (5% (*w*/*v*) milk powder in tris-buffered saline with 0.1% Tween 20 (TBST)) and incubated overnight in at 4 °C in block buffer with either the primary antibody targeting cMyc (#9402–1:2000 *v*/*v*; Cell Signaling, Leiden, the Netherlands) or the loading control vinculin (#4650–1:1000 *v*/*v*, Cell Signaling). The next day, blots were washed in TBST and incubated for 1 h in block buffer with the secondary antibody (#7074S-1:2000 *v*/*v*; Cell Signaling). Blots were washed again and treated with SuperSignal West Femto (Thermo Fisher Scientific) to visualize protein bands. Blots were analyzed with a ChemiDoc Touch Imaging system (Bio-Rad) and band intensities were normalized to vinculin.

### 2.7. Statistics

Statistical analysis was performed with IBM SPSS statistics version 25.0 (SPSS Inc., Chicago, IL, USA). Data were tested for normality with the Shapiro-Wilk test and for equal variance with Levene’s test (α = 0.05). Normally distributed data with equal variance was tested with an one-way analysis of variance (ANOVA) with a Dunnet’s post hoc test (HFD was the control group for in vivo data, TGF-β condition was the control group for in vitro data). The Kruskal–Wallis test with a Mann–Whitney post hoc test was performed for data that were not normally distributed or had equal variance.

## 3. Results

### 3.1. Butyrate Attenuates HFD-Induced Obesity, Improves Metabolic Risk Factors, and Reduces Adipose Tissue Inflammation

Long-term HFD feeding over 38 weeks significantly increased body weight relative to chow, while energy intake was comparable in the HFD and chow group. The observed increase in body weight with HFD was associated with a higher food efficiency (body weight gain per kcal consumed) in the HFD group (Table 1). Treatment with BU reversed these HFD-induced effects as observed by a decrease in body weight and energy intake (Table 1). Moreover, food efficiency was lowered compared with HFD, indicating that BU has additional positive effects on body weight, independent of energy intake (Table 1). HFD feeding increased plasma cholesterol and triglycerides, which were unaffected by BU. Glucose levels were comparable in all groups, while fasting insulin concentrations were strongly increased by HFD feeding and significantly lowered with BU, reaching insulin concentrations below those observed in chow (Table 1).

**Figure 1:**
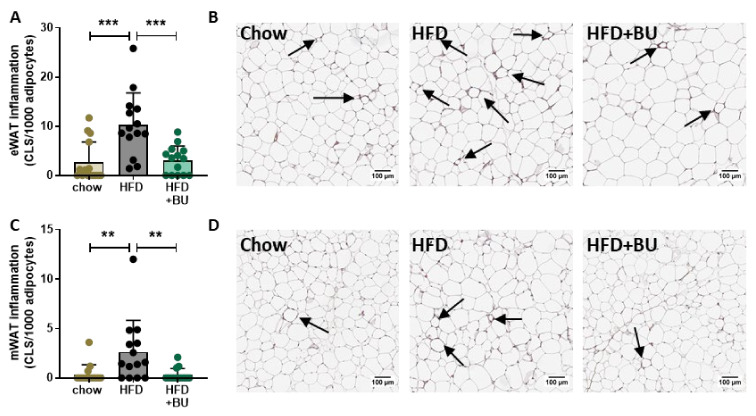
Butyrate reduced white adipose tissue inflammation (WAT). The number of inflammatory crown-like structures (indicated with arrows) were scored in (**A**) epidydimal WAT (eWAT), and shown in (**B**) the representative images of eWAT inflammation. Inflammation was also scored in (**C**) mesenteric WAT (mWAT and shown in (**D**)) the respective images representing mWAT inflammation. Data are presented as mean ± SD, ** *p* < 0.01, *** *p* < 0.001 vs. the HFD control group.

The observed increase in fat mass with HFD was accompanied by alterations in plasma adipokine concentrations, i.e., leptin was increased and adiponectin decreased. BU reduced plasma leptin concentrations with no effect on adiponectin (Table 1), suggesting a beneficial effect on adipose tissue. Quantitative histological analysis of inflammation in epidydimal and mesenteric white adipose tissue (WAT) depots demonstrated that the number of crown-like structures (CLS) was increased by HFD in both WAT depots (Figure 1A–D). BU lowered the number of CLS in both WAT depots, in line with the reduction in proinflammatory plasma leptin level.

### 3.2. Butyrate Did Not Affect the HFD-Induced Increase in Gut Permeability or Changes in Microbiota Composition

**Figure 2:**
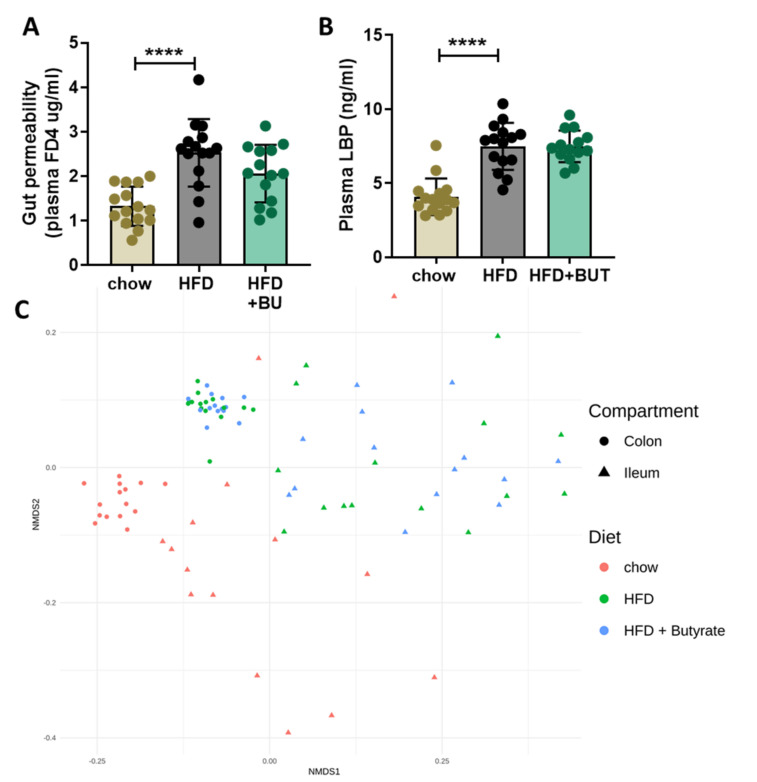
Butyrate does not affect gut permeability and microbiota composition. (**A**) Functional gut permeability fluorescein isothiocyanate-labeled dextran (FD4) assay. (**B**) Plasma LPS binding protein (LBP) concentrations. Data are presented as mean ± SD, **** *p* < 0.0001 vs. the HFD control group. (**C**) 16S rRNA gene analysis was performed to study the microbiota composition in fecal and mucosal compartments of both the colon and ileum. These changes in microbiota composition were visualized by non-metric multidimensional scaling (NMDS) using the Bray–Curtis index, in which every dot represents the microbiota composition of one mouse and the distance between dots represent how (dis)similar the microbiota composition is between these mice.

To determine whether these metabolic improvements with BU may be associated with beneficial effects in the gut, we performed a functional gut permeability test, measured plasma LPS-binding protein (LPB) concentrations, and performed a 16S rRNA microbiota composition analysis. Gut permeability was increased with HFD and unaffected by BU (Figure 2A). Similarly, plasma concentrations of the LPB were increased with HFD and not altered by BU (Figure 2B). Microbiota composition analysis was performed in the mucosal compartments of both the colon and ileum. Permutation tests which enable the statistical testing of differences in microbiota composition between experimental groups showed that HFD feeding altered both ileum (*p* = 0.002) and colon (*p* = 0.002) colonization relative to chow, as presented in Figure 2C. An overview of the significantly different genera between the experimental groups are provided in Supplementary Figures S1–S3. BU did not affect microbiota composition compared with HFD in colon (*p* = 0.234) and was affected only the abundance of three genera in ileum (*p* = 0.048), as visualized in Figure 2C by the extensive overlap between HFD and butyrate-fed mice.

Taken together, in the context of an obese phenotype with pronounced hyperinsulinemia, hyperlipidemia, WAT, and gut dysfunction, BU decreased body weight, WAT inflammation, and reversed hyperinsulinemia. All of these beneficial BU effects seem to be independent of the gut microbiota composition or gut permeability as these were largely unaffected by BU.

### 3.3. Butyrate Protected Mice against NASH Development

**Figure 3:**
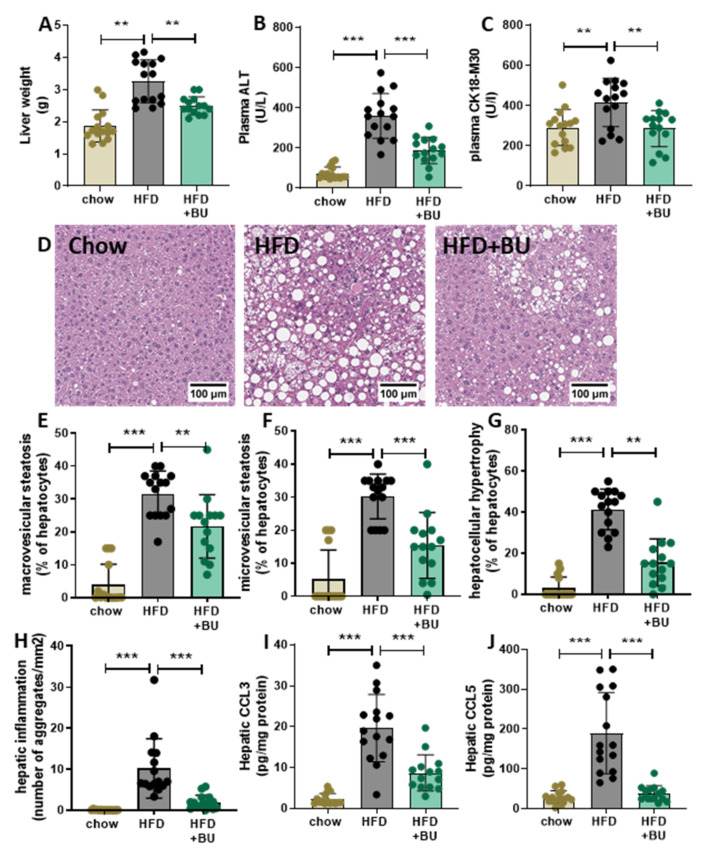
Butyrate reduced plasma liver damage markers, steatosis, and hepatic inflammation in 38-week HFD-fed animals. (**A**) Liver weight, (**B**) plasma liver damage maker ALT, and (**C**) CK18-m30. (**D**) Representative images of hematoxylin-eosin stained liver sections analyzed for (**E**) macrovesicular steatosis, (**F**) microvesicular steatosis, (**G**) hepatocellular hypertrophy, and (**H**) hepatic inflammation, supported by hepatic chemokine concentrations (**I**) CCL3 and (**J**) CCL5, expressed per mg protein. Data are presented as mean ± SD, ** *p* < 0.01, *** *p* < 0.001 vs. the HFD control group.

Liver weight was increased in control mice on an HFD, whereas BU-fed mice were protected from the HFD-induced liver weight gain (Figure 3A). Levels of the circulating liver damage markers ALT and CK18-M30 were increased with HFD and were lowered by BU (Figure 3B,C). To investigate the effect on the liver in more detail, liver cross sections were analyzed for histopathological features of NASH (Figure 3D). Steatosis was hardly present in the chow-fed mice, whereas HFD-fed mice developed pronounced steatosis with equal induction of macrovesicular (Figure 3E) and microvesicular steatosis (Figure 3F). BU significantly reduced steatosis observed by a similar reduction in both macrovesicular and microvesicular steatosis. Consistent with this, HFD increased the cross-sectional area covered with abnormally enlarged hepatocytes, which was attenuated with BU (Figure 3G). Hepatic inflammation was practically absent in chow-fed mice, and HFD strongly increased the number of inflammatory aggregates. BU significantly suppressed the number of inflammatory aggregates (Figure 3H). These anti-inflammatory effects were confirmed by analyzing hepatic chemokine concentrations. HFD increased hepatic CCL3 (MIP1a) and CCL5 (RANTES), which were significantly decreased with butyrate (Figure 3I,J). HFD feeding also significantly increased CXCL1 (KC) and CXCL10 (IP-10), which were not affected by butyrate (Supplementary Figure S4). Altogether, HFD feeding resulted in a pronounced increase in steatosis and inflammation in the liver, which was strongly attenuated by BU.

### 3.4. Butyrate Reduced Liver Fibrosis

**Figure 4:**
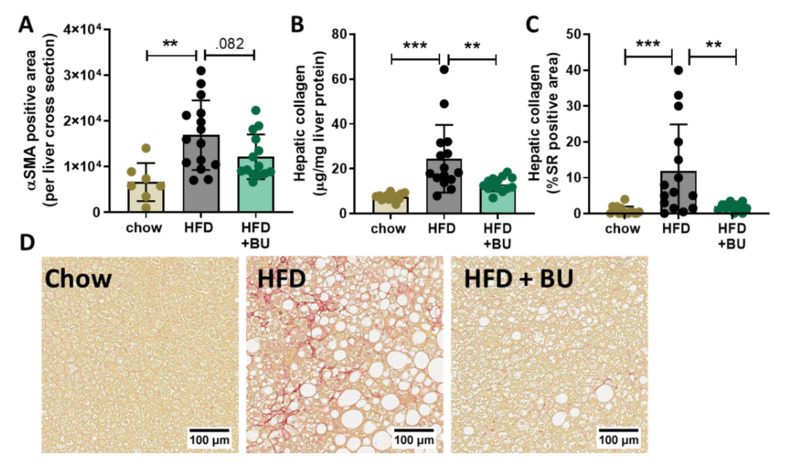
Butyrate decreased liver fibrosis in 38-week HFD-fed animals. (**A**) Alpha-smooth muscle cell actin (αSMA) immunoreactive positive area in liver cross sections, (**B**) hepatic collagen content measured in liver homogenates, (**C**) Sirius-red (SR) positive area of (**D**) Sirius-red stained cross-sections (representative images). Data are presented as mean ± SD, ** *p* < 0.01, *** *p* < 0.001 vs. the HFD control group.

HFD feeding strongly increased the alpha smooth muscle cell actin (αSMA)-positive area in liver cross sections, which is a marker for activated hepatic stellate cell (HSC), the main collagen-producing cells in the liver. BU tended to lower this HFD-induced αSMA positive area (Figure 4A). In line with this data, HFD significantly increased the hepatic collagen content as demonstrated in freshly prepared liver homogenates (Figure 4B). In BU-fed mice hepatic collagen content was significantly lower, indicating that BU-fed mice were protected against HFD-induced liver fibrosis (Figure 4B). This was confirmed by histopathological analysis of fibrosis, which showed that HFD increased the Sirius-red-positive area and BU fully blunted this induction (Figure 4C). Development of liver fibrosis was accompanied by significant increases in circulating tissue inhibitor of metalloproteinase 1 (TIMP1) and ECM remodeling markers of pericellular fibrosis, PRO-C4 and C6M (Supplementary Table S1). BU did not significantly affect TIMP1 or ECM turnover biomarkers.

Thus, BU-fed mice were not only protected against NASH development but also showed strongly reduced fibrosis, which was not reflected by the measured circulating ECM remodeling markers.

### 3.5. Butyrate Directly Reduced TGF-β-Induced Collagen Production in Primary Human Hepatic Stellate Cells

**Figure 5:**
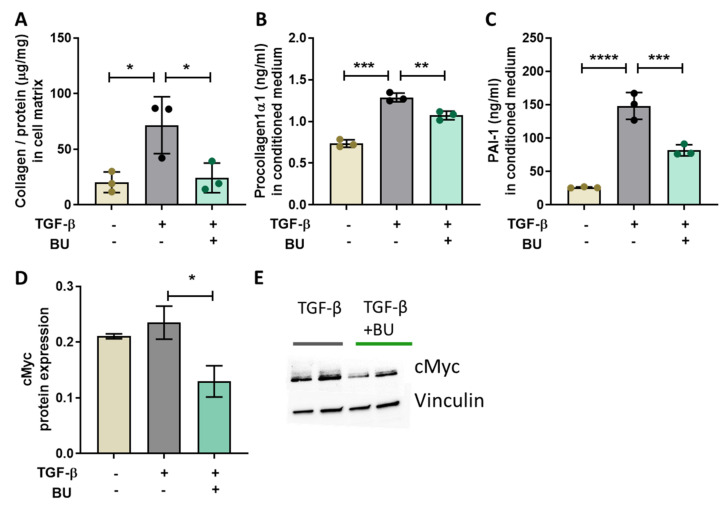
Butyrate directly decreased TGF-β induced fibrosis in primary human hepatic stellate cells. (**A**) Collagen content in the cell matrix, (**B**) procollagen1a1 protein expression, and (**C**) PAI-1 protein expression in conditioned medium. Western blot analysis was used to determine (**D**) cMyc protein expression normalized for vinculin protein expression. (**E**) Representative images of the western blot bands. Data are presented as mean ± SD, * *p* < 0.05, ** *p* < 0.01, *** *p* < 0.001, **** *p* <0.0001 compared with the TGF-β control group.

To further elucidate the mechanisms underlying these antifibrotic effects, we studied BU treatment effects on collagen production by hepatic stellate cells (HSC) in vitro, followed by a transcriptomics analysis to assess modulation of underlying pathways and regulators. After defining an optimal dose for BU in cell viability assays (Supplementary Figure S5), TGF-β-stimulated primary human HSC treated with 1 mM BU demonstrated a consistent strong reduction of TGF-β-induced collagen deposition showing that BU has direct anti-fibrotic effects in HSC (Figure 5A). This was further confirmed by reduced procollagen1α1 levels in conditioned culture medium from BU-treated cells, suggesting that the observed collagen decrease is a result of reduced collagen synthesis (Figure 5B). In addition, BU significantly attenuated TGF-β-induced protein expression of the stellate cell activation marker PAI1, which demonstrates that BU inhibited activation of HSC into proliferative fibrogenic myofibroblasts (Figure 5C). In line with this, a reduction was observed in gene expression of HSC activation markers (e.g., ACTA2 encoding for αSMA, SERPINE1 encoding for PAI1, ELN) (Table 2). These anti-fibrotic effects were found to be specific and dose-dependent for the SCFA butyrate, since other SCFAs did not affect collagen deposition in TGF-β-stimulated HSC (Supplementary Figure S6).

To gain mechanistic insight into these anti-fibrotic effects of BU, we performed a subsequent upstream regulator analysis on the gene expression data. As expected, TGF-β strongly activated the TGF-β receptor and canonical pathway signaling downstream of the TGF-β receptor (Table 3). Although a number of these factors were significantly enriched by BU treatment, most of the upstream regulators did not reach the cut-off value for consistent activation or inactivation—indicating that attenuation of TGF-β induced fibrosis by BU is not mediated via modification of the canonical TGF-β signaling pathway (Table 3). Importantly, TGF-β also activated non-canonical pathways downstream of the TGF-β receptor (involving ERK, JNK/P38, Rho-like GTPases and PI3K/AKT). BU did not affect the pathways via ERK and JNK/P38, but consistently deactivated the non-canonical signaling routes mediated by Rho-like GTPases and PI3K/AKT/MYC. Of note, BU did not exert its effect on one particular component of these pathways, but tended to simultaneously inactivate multiple of its components. Furthermore, BU also reduced TGF-β-induced activation of the Hippo signaling pathway (YAP/TAZ), a critical pathway in liver fibrosis [30]. To confirm our observations on protein level we performed a western blot analysis on cMyc protein expression. In line with the upstream regulator analysis we found that TGF-β non-significantly increased cMyc expression, whereas butyrate significantly decreased cMyc protein expression (Figure 5D,E).

In conclusion, BU specifically and dose-dependently decreased collagen synthesis in TGF-β stimulated HSC by attenuating activation of HSC into proliferative fibrogenic myofibroblasts. Gene expression analysis indicated that BU deactivated non-canonical TGF-ß signaling pathways involving Rho-like GTPases and PI3K/AKT/MYC, confirmed by western blot analysis for cMyc protein expression, providing a rationale for its antifibrotic effects.

## 4. Discussion

The objective of this long-term BU supplementation study was to investigate potential anti-fibrotic effects of BU in the context insulin-resistance-associated obesity. Indeed, mice fed BU for 38 weeks were protected against obesity-associated WAT inflammation, hyperinsulinemia, and NASH, and BU strongly suppressed development of liver fibrosis. These anti-fibrotic effects were supported by in vitro experiments in HSC demonstrating that BU directly and dose-dependently inhibited TGF-β induced collagen production.

This long-term dietary treatment study of 38 weeks allowed the examination of NASH-associated liver fibrosis. We demonstrated that BU-fed mice were protected against liver fibrosis development as hepatic collagen content and Sirius-red-positive area were decreased. The anti-fibrotic effects of BU observed in vivo were further substantiated by a dose-dependent reduction in collagen production by BU in an in vitro fibrosis model using TGF-β induced primary human HSC. Quiescent HSC comprise 10% of the liver and, in response to chronic liver injury, HSC will be activated and expand to approximately 15% of the total liver cells [31], concomitant with a major upregulation of hepatic collagen production. Importantly, only the SCFA BU—but not other SCFAs—lowered collagen deposition, indicating that the anti-fibrotic effect is specific for BU. NGS gene expression analysis demonstrated that HSC activation was attenuated with butyrate (e.g., SERPINE1 encoding for PAI1 and ACTA2 encoding for αSMA) [32,33], supported by a decrease in PAI1 protein expression in the HSC, and also in vivo by the αSMA staining in the livers of the mice. Subsequent pathway and upstream regulator analysis on the NGS data showed that BU did not affect canonical TGF-β signaling, but rather inactivated specific non-canonical pathways Rho-like GTPases and PI3K/AKT. RhoA and Rock GTPases promote nuclear localization and activation of YAP/TAZ to stimulate fibrosis [34], as observed in TGF-β stimulated HSC, and BU counteracted this effect. Suppression of YAP/TAZ activation by BU may be of particular importance because YAP/TAZ has been shown to sense ECM stiffness and to promote ECM production in a self-sustaining feedforward loop [34]. Interestingly, it was very recently shown that PAI-1 protein expression is mainly determined by the YAP/TAZ signaling pathway [35]. Therefore, the reduction of PAI-1 protein expression shown with butyrate in the present study confirms the predicted observed reduction in the Hippo signaling pathway (YAP/TAZ signaling) on the protein level. Also of note is the pronounced deactivation of MYC upon BU treatment, as confirmed by a decrease in cMyc protein expression with western blot analysis, since this has been demonstrated to be a marker of liver fibrosis [36] and overexpression of MYC alone induces HSC activation [37].

The observed NASH-attenuating effects are in line with findings in other rodent models that rely on the use of supraphysiological levels of cholesterol to induce liver inflammation [38,39] or methionine-choline-deficient diet models [40], which both focus on a very specific aspect of disease development. In this study, we used a model that makes use of translational dietary conditions (with a macronutrient composition and a natural cholesterol content similar to human diets) [17] and reflects the multiple organ dysfunction that is characteristic of NASH in humans [3,18]. Under these translational experimental conditions, we demonstrated that BU supplementation protects against diet-induced obesity, WAT inflammation, hyperinsulinemia, NASH, and liver fibrosis. These findings with BU cannot be merely ascribed to a reduction in caloric intake as food efficiency calculations showed that BU decreased the body weight increase per consumed calorie. Li et al. demonstrated that BU modestly increased fat oxidation in metabolic organs such as brown adipose tissue and muscle tissue [16], which may contribute to the observed weight loss independent of food intake. In line with this, the observed anti-fibrotic effects of BU can also not be linked directly to the reduction in energy intake, as demonstrated by a comparison of historical data from HFD-fed Ldlr-/-.Leiden control mice (Supplementary Figure S7)—which showed no difference in fibrosis development between mice with ‘normal’ energy intake (comparable to the HFD control group in the current study) and mice with ‘low’ energy intake (comparable to the HFD+BU group in the current study).

The pronounced hepatoprotective effects by BU seem to be mediated independent of the gut, as BU did not affect gut permeability or microbiota composition. It is possible that BU in the current study did not reach the colon, since absorption of SCFAs such as BU already takes place in the stomach [41] and ileum [42,43,44]. In line with this notion, studies using higher BU supplementation concentrations did report changes in fecal [9,40] or cecal [16] microbiota composition. Changes in microbiota composition are typically based on the fecal microbiota composition since it is easily accessible. However, we have previously shown that the colon mucosal site has better predictive value for metabolic dysfunction and NAFLD development [4]. Moreover, a reasonable assumption is that bacteria in the mucosal layer are in closer proximity to the gut barrier and therefore in more direct contact with the host than the fecal microbiota. Changes in functional gut permeability with BU in the context of metabolic overload induced NASH have not been studied yet to our knowledge, though increased gut barrier protein expression with butyrate has been reported [9]. Attenuation of gut permeability has been studied in the context of acute infections [45], stroke [46], and atherosclerosis models [47]. The experimental conditions employed in the present study (i.e., long-term HFD treatment with chronic low-grade inflammation) deviate from these models and it is possible that in other (more severe conditions) BU has different effects. Additionally, differences may be attributable to the higher butyrate dosages used in other studies.

BU attenuated WAT inflammation associated with an improvement in adipokine secretion, i.e., lowering of circulating proinflammatory leptin in line with previous observations [48]. Ikejima et al. demonstrated that sinusoidal endothelial cells and Kupffer cells both express functional leptin receptors and, when studied in vitro, respond to leptin with increased expression of TGF-β [49], the main pro-fibrotic cytokine. Furthermore, leptin has also been described to directly stimulate HSC activation in vitro [50]. The observed anti-inflammatory effect in WAT and lowered circulating leptin levels in the current study could therefore also indirectly have contributed to the attenuation of hepatic inflammation and liver fibrosis with BU.

A limitation of the current in vivo study is that it does not allow investigation of body weight-independent effects of BU. Nevertheless, the food-efficiency calculations and results from the in vitro experiments do provide an indication that BU has additional (direct) beneficial effects on top of the observed anti-obesogenic effects. These may be further investigated in future studies that include a pair-fed control group. In addition, as mentioned above, butyrate that is supplemental to the diet can already be absorbed in the upper intestinal tract and its effects are therefore not directly comparable to the effects of microbially produced BU, which is absorbed in the colon (and most likely becomes available systemically at lower doses than those used herein). However, the route of administration chosen here does allow direct and specific investigation of the effects of BU, while studies using fermentable fibers to increase microbial butyrate production indirectly will always be confounded by other fiber-induced effects.

In conclusion, we demonstrate that in the context of an obese phenotype with multiple-organ dysfunction and insulin resistance, BU protected mice against diet-induced NASH and liver fibrosis development. The anti-fibrotic effects of BU may at least in part be explained by direct inhibition of collagen synthesis in hepatic stellate cells involving suppression of specific non-canonical TGF-β signaling pathways Rho-like GTPases and PI3K/AKT, and other important pro-fibrotic regulators (e.g., YAP/TAZ, MYC).

## Figures and Tables

**Table 1:** Body composition and metabolic risk factors.

	Chow	HFD	HFD + BU
Body weight (g)	42.4 ± 6.3 ***	56.5 ± 3.9	45.3 ± 4.4 ***
Average energy intake (kcal/mouse/day)	12.7 ± 0.5	13.4 ± 0.3	11.6 ± 0.5 **
Average food efficiency (mg BW gain/kcal)	3.85 ± 1.72 ***	7.54 ± 1.14	5.73 ± 0.86 ***
Cholesterol (mM)	9.3 ± 2.2 ***	34.4 ± 6.4	35.7 ± 7.5
Triglycerides (mM)	1.7 ± 0.6 ***	5.5 ± 2.1	6.3 ± 2.5
Glucose (mM)	7.1 ± 1.1	6.6 ± 0.9	6.6 ± 1.2
Insulin (ng/mL)	9.3 ± 16.4 *	20.3 ± 11.1	8.1 ± 4.2 *
Leptin (ng/mL)	25.3 ± 13.3 ***	59.2 ± 16.0	39.3 ± 9.5 ***
Adiponectin (ug/mL)	7.2 ± 1.2 *	6.1 ± 1.6	6.2 ± 0.8

BW = body weight. Data are presented as mean ± SD, * *p* < 0.05, ** *p* < 0.01, *** *p* < 0.001 vs. the HFD control group.

**Table 2:** Gene expression analysis in primary human stellate cells.

	TGF-β vs. Unstimulated		BU+TGF-β vs. TGF-β	
Gene Name:	2logR	*p*-Value	2logR	*p*-Value
**Myofibroblast markers**				
ACTA2	4.28	0.000	−1.15	0.000
SERPINE1	2.03	0.000	−0.37	0.000
ELN	2.13	0.000	−1.40	0.000
CDH11	1.43	0.000	−1.45	0.000
DES	0.70	0.007	1.62	0.000
VIM	−0.02	0.682	0.79	0.000
PALLD	2.29	0.000	−0.27	0.000
TPM1	3.24	0.000	0.13	0.002
CFL1	0.62	0.000	−0.26	0.000
COL1A1	0.63	0.000	−0.05	0.402

Changes in gene expression of myofibroblast markers indicative for hepatic stellate cell activation. Gene expression changes are expressed in 2log fold-change (2logR), red indicates upregulation in gene expression, green indicates downregulation, and significant changes are marked in grey.

**Table 3:** Upstream regulator analysis in primary human stellate cells.

	TGF-β vs. Unstimulated		BU+TGF-β vs. TGF-β	
Upstream Regulator:	Z-Score	*p*-Value	Z-Score	*p*-Value
**TGF-β signaling**				
TGFB1	9.7	0.000	1.5	0.000
TGFB2	5.1	0.000	−0.4	0.002
TGFB3	5.6	0.000	−0.7	0.000
TGFBR1	2.6	0.000	−0.2	0.030
TGFBR2	1.8	0.000	−0.1	0.000
TGFBR3	−1.0	0.026	N/A	N/A
**Canonical TGF-β signaling**				
SMAD1	0.4	0.000	0.7	0.003
SMAD2	4.5	0.000	N/A	1.000
SMAD3	2.8	0.000	2.0	0.014
SMAD4	3.5	0.000	1.3	0.002
SMAD7	−1.5	0.000	−0.9	0.002
SMAD2/3/4	2.6	0.001	N/A	1.000
SMAD1/5/9	N/A	1.000	N/A	0.022
**Non-canonical TGF- β signaling**				
**via ERK**				
RAS	2.9	0.000	0.9	0.000
RAF	0.2	0.000	0.5	0.000
MAP2K1	1.6	0.000	2.1	0.000
ERK	3.0	0.000	3.1	0.000
CAV1	1.3	0.000	1.6	0.000
**via JNK/p38**				
MAP3K7	0.6	0.000	N/A	1.000
JNK	3.4	0.000	0.0	0.000
MAPK11	0.6	0.030	2.4	0.120
MAPK13	−0.8	0.039	1.3	0.037
MAPK14	1.4	0.000	2.7	0.000
AP1	2.2	0.000	2.7	0.026
**via Rho-like GTPases**				
RHOA	1.2	0.001	−1.0	0.001
ROCK	1.5	0.000	−0.9	0.000
CDC42	N/A	1.000	N/A	1.000
RAC1	2.7	0.000	N/A	1.000
PAK1	1.4	0.006	−1.0	0.019
PAK2	−0.3	0.000	−1.6	0.006
**via PI3K/AKT**				
AKT	3.3	0.000	0.2	0.000
PTEN	−2.0	0.000	1.2	0.000
GSK3B	0.6	0.011	−1.1	0.042
MDM2	N/A	1.000	−0.7	0.008
MYC	2.2	0.000	−6.2	0.000
MTORC1	0.7	0.003	N/A	1.000
**HIPPO signaling**				
YAP1	2.5	0.000	−2.1	0.000
WWTR1	0.8	0.000	−1.8	0.000

The activity of an upstream regulator was calculated based on gene expression changes of all downstream target genes. A Z-score < −2 indicates inhibition of the respective regulator or pathway (green color) and Z > 2 indicates activation (red color). The *p*-value < 0.05 in grey indicates significant enrichment of the target genes downstream of a regulator, i.e., that more downstream genes are affected than can be expected by chance. N/A indicates an insufficient number of differentially expressed genes to predict the activation state of an upstream regulator.

## Data Availability

The transcriptomics data are available on Gene Expression Omnibus (GEO), data set GSE179395.

## Supplementary Materials

The following are available online at https://www.mdpi.com/article/10.3390/biomedicines9121954/s1, Figure S1: List of genera in the colon mucosa, Figure S2: Genera expressed in the mucosal layer of the ileum, Figure S3: Genera expressed in the mucosal layer of the ileum, Figure S4: Hepatic chemokine concentrations, Table S1: Circulating extracellular matrix remodeling markers, Figure S5: Cytotoxicity of different BU dosages, Figure S5: Effect of short chain fatty acids acetate (AC), caproate (CA) and butyrate (BU) on TGF-β induced fibrosis in primary human hepatic stellate cells, Figure S7 Association between energy intake and liver fibrosis.

## References

[1] Pelusi S., Cespiati A., Rametta R., Pennisi G., Mannisto V., Rosso C., Baselli G., Dongiovanni P., Fracanzani A.L., Badiali S. (2019). Prevalence and Risk Factors of Significant Fibrosis in Patients with Nonalcoholic Fatty Liver without Steatohepatitis. Clin. Gastroenterol. Hepatol..

[2] Vilar-Gomez E., Calzadilla-Bertot L., Wai-Sun Wong V., Castellanos M., Aller-de la Fuente R., Metwally M., Eslam M., Gonzalez-Fabian L., Alvarez-Quiñones Sanz M., Conde-Martin A.F. (2018). Fibrosis Severity as a Determinant of Cause-Specific Mortality in Patients with Advanced Nonalcoholic Fatty Liver Disease: A Multi-National Cohort Study. Gastroenterology.

[3] Brown G.T., Kleiner D.E. (2016). Histopathology of nonalcoholic fatty liver disease and nonalcoholic steatohepatitis. Metabolism.

[4] Gart E., Lima E.S., Schuren F., De Ruiter C.G.F., Attema J., Verschuren L., Keijer J., Salic K., Morrison M.C. (2019). Diet-Independent Correlations between Bacteria and Dysfunction of Gut, Adipose Tissue, and Liver: A Comprehensive Microbiota Analysis in Feces and Mucosa of the Ileum and Colon in Obese Mice with NAFLD. Int. J. Mol. Sci..

[5] Loomba R., Friedman S.L., Shulman G.I. (2021). Mechanisms and disease consequences of nonalcoholic fatty liver disease. Cell.

[6] Baiocchini A., Montaldo C., Conigliaro A., Grimaldi A., Correani V., Mura F., Ciccosanti F., Rotiroti N., Brenna A., Montalbano M. (2016). Extracellular matrix molecular remodeling in human liver fibrosis evolution. PLoS ONE.

[7] Dewidar B., Soukupova J., Fabregat I., Dooley S. (2015). TGF-β in Hepatic Stellate Cell Activation and Liver Fibrogenesis: Updated. Curr. Pathobiol. Rep..

[8] Zhang Y.E. (2009). Non-Smad pathways in TGF-β signaling. Cell Res..

[9] Zhou D., Pan Q., Xin F.Z., Zhang R.N., He C.X., Chen G.Y., Liu C., Chen Y.W., Fan J.G. (2017). Sodium butyrate attenuates high-fat diet-induced steatohepatitis in mice by improving gut microbiota and gastrointestinal barrier. World J. Gastroenterol..

[10] Sun B., Jia Y., Hong J., Sun Q., Gao S., Hu Y., Zhao N., Zhao R. (2018). Sodium butyrate ameliorates high-fat diet-induced NAFLD through PPARα-mediated activation of β oxidation and suppression of inflammation. J. Agric. Food Chem..

[11] Canfora E.E., Jocken J.W., Blaak E.E. (2015). Short-chain fatty acids in control of body weight and insulin sensitivity. Nat. Rev. Endocrinol..

[12] Mattace Raso G., Simeoli R., Russo R., Iacono A., Santoro A., Paciello O., Ferrante M.C., Canani R.B., Calignano A., Meli R. (2013). Effects of Sodium Butyrate and Its Synthetic Amide Derivative on Liver Inflammation and Glucose Tolerance in an Animal Model of Steatosis Induced by High Fat Diet. PLoS ONE.

[13] Arnoldussen I.A.C., Wiesmann M., Pelgrim C.E., Wielemaker E.M., Van Duyvenvoorde W., Amaral-Santos P.L., Verschuren L., Keijser B.J.F., Heerschap A., Kleemann R. (2017). Butyrate restores HFD-induced adaptations in brain function and metabolism in mid-adult obese mice. Int. J. Obes..

[14] Bouter K.E.C., Bakker G.J., Levin E., Hartstra A.V., Kootte R.S., Udayappan S.D., Katiraei S., Bahler L., Gilijamse P.W., Tremaroli V. (2018). Differential metabolic effects of oral butyrate treatment in lean versus metabolic syndrome subjects article. Clin. Transl. Gastroenterol..

[15] Cerdó T., García-Santos J.A., Bermúdez M.G., Campoy C. (2019). The role of probiotics and prebiotics in the prevention and treatment of obesity. Nutrients.

[16] Li Z., Yi C.X., Katiraei S., Kooijman S., Zhou E., Chung C.K., Gao Y., Van Den Heuvel J.K., Meijer O.C., Berbée J.F.P. (2018). Butyrate reduces appetite and activates brown adipose tissue via the gut-brain neural circuit. Gut.

[17] Mueller A.M., Kleemann R., Gart E., van Duyvenvoorde W., Verschuren L., Caspers M., Menke A., Krömmelbein N., Salic K., Burmeister Y. (2021). Cholesterol Accumulation as a Driver of Hepatic Inflammation Under Translational Dietary Conditions Can Be Attenuated by a Multicomponent Medicine. Front. Endocrinol..

[18] Morrison M.C., Verschuren L., Salic K., Verheij J., Menke A., Wielinga P.Y., Iruarrizaga-Lejarreta M., Gole L., Yu W., Turner S. (2018). Obeticholic Acid Modulates Serum Metabolites and Gene Signatures Characteristic of Human NASH and Attenuates Inflammation and Fibrosis Progression in Ldlr-/-.Leiden Mice. Hepatol. Commun..

[19] Van Koppen A., Verschuren L., van den Hoek A.M., Verheij J., Morrison M.C., Li K., Nagabukuro H., Costessi A., Caspers M.P.M., van den Broek T.J. (2018). Uncovering a Predictive Molecular Signature for the Onset of NASH-Related Fibrosis in a Translational NASH Mouse Model. Cell. Mol. Gastroenterol. Hepatol..

[20] Van den Hoek A.M., de Jong J.C.B.C., Worms N., van Nieuwkoop A., Voskuilen M., Menke A.L., Lek S., Caspers M.P.M., Verschuren L., Kleemann R. (2021). Diet and exercise reduce pre-existing NASH and fibrosis and have additional beneficial effects on the vasculature, adipose tissue and skeletal muscle via organ-crosstalk. Metabolism.

[21] Nielsen M.J., Nedergaard A.F., Sun S., Veidal S.S., Larsen L., Zheng Q., Suetta C., Henriksen K., Christiansen C., Karsdal M.A. (2013). The neo-epitope specific PRO-C3 ELISA measures true formation of type III collagen associated with liver and muscle parameters. Am. J. Transl. Res..

[22] Leeming D.J., Nielsen M.J., Dai Y., Veidal S.S., Vassiliadis E., Zhang C., He Y., Vainer B., Zheng Q., Karsdal M.A. (2012). Enzyme-linked immunosorbent serum assay specific for the 7S domain of collagen type IV (P4NP 7S): A marker related to the extracellular matrix remodeling during liver fibrogenesis. Hepatol. Res..

[23] Sand J.M., Larsen L., Hogaboam C., Martinez F., Han M. (2013). Larsen, M.R.; Nawrocki, A.; Zheng, Q.; Karsdal, M.A.; Leeming, D.J. MMP Mediated Degradation of Type IV Collagen Alpha 1 and Alpha 3 Chains Reflects Basement Membrane Remodeling in Experimental and Clinical Fibrosis-Validation of Two Novel Biomarker Assays. PLoS ONE.

[24] Skovgård Veidal S., Karsdal M.A., Vassiliadis E., Nawrocki A., Larsen M.R., Nguyen H.T., Hägglund P., Luo Y., Zheng Q., Vainer B. (2011). MMP Mediated Degradation of Type VI Collagen Is Highly Associated with Liver Fibrosis-Identification and Validation of a Novel Biochemical Marker Assay. PLoS ONE.

[25] Galarraga M., Campión J., Munõz-Barrutia A., Boqué N., Moreno H., Martínez J.A., Milagro F., Ortiz-de-Solórzano C. (2012). Adiposoft: Automated software for the analysis of white adipose tissue cellularity in histological sections. J. Lipid Res..

[26] Liang W., Menke A.L., Driessen A., Koek G.H., Lindeman J.H., Stoop R., Havekes L.M., Kleemann R., Van Den Hoek A.M. (2014). Establishment of a general NAFLD scoring system for rodent models and comparison to human liver pathology. PLoS ONE.

[27] Love M.I., Huber W., Anders S. (2014). Moderated estimation of fold change and dispersion for RNA-seq data with DESeq2. Genome Biol..

[28] Krämer A., Green J., Pollard J., Tugendreich S. (2014). Causal analysis approaches in Ingenuity Pathway Analysis. Bioinformatics.

[29] Gart E., Salic K., Morrison M.C., Caspers M., Van Duyvenvoorde W., Heijnk M., Giera M., Bobeldijk-Pastorova I., Keijer J., Storsve A.B. (2021). Krill Oil Treatment Increases Distinct PUFAs and Oxylipins in Adipose Tissue and Liver and Attenuates Obesity-Associated Inflammation via Direct and Indirect Mechanisms. Nutrients.

[30] Mannaerts I., Leite S.B., Verhulst S., Claerhout S., Eysackers N., Thoen L.F.R., Hoorens A., Reynaert H., Halder G., Van Grunsven L.A. (2015). The Hippo pathway effector YAP controls mouse hepatic stellate cell activation. J. Hepatol..

[31] Kisseleva T., Cong M., Paik Y.H., Scholten D., Jiang C., Benner C., Iwaisako K., Moore-Morris T., Scott B., Tsukamoto H. (2012). Myofibroblasts revert to an inactive phenotype during regression of liver fibrosis. Proc. Natl. Acad. Sci. USA.

[32] Ghosh A.K., Vaughan D.E. (2012). PAI-1 in Tissue Fibrosis. J. Cell. Physiol..

[33] Noguchi R., Kaji K., Namisaki T., Moriya K., Kawaratani H., Kitade M., Takaya H., Aihara Y., Douhara A., Asada K. (2020). Novel oral plasminogen activator inhibitor.1 inhibitor TM5275 attenuates hepatic fibrosis under metabolic syndrome via suppression of activated hepatic stellate cells in rats. Mol. Med. Rep..

[34] Noguchi S., Saito A., Nagase T. (2018). YAP/TAZ Signaling as a Molecular Link between Fibrosis and Cancer. Int. J. Mol. Sci..

[35] Higgins C.E., Tang J., Higgins S.P., Gifford C.C., Mian B.M., Jones D.M., Zhang W., Costello A., Conti D.J., Samarakoon R. (2021). The Genomic Response to TGF-β1 Dictates Failed Repair and Progression of Fibrotic Disease in the Obstructed Kidney. Front. Cell Dev. Biol..

[36] Shen Y., Miao N., Wang B., Xu J., Gan X., Xu D., Zhou L., Xue H., Zhang W., Yang L. (2017). c-Myc promotes renal fibrosis by inducing integrin αv-mediated transforming growth factor-β signaling. Kidney Int..

[37] Nevzorova Y.A., Hu W., Cubero F.J., Haas U., Freimuth J., Tacke F., Trautwein C., Liedtke C. (2013). Overexpression of c-myc in hepatocytes promotes activation of hepatic stellate cells and facilitates the onset of liver fibrosis. Biochim. Biophys. Acta - Mol. Basis Dis..

[38] Baumann A., Jin C.J., Brandt A., Sellmann C., Nier A., Burkard M., Venturelli S., Bergheim I. (2020). Oral supplementation of sodium butyrate attenuates the progression of non-alcoholic steatohepatitis. Nutrients.

[39] Jin C.J., Sellmann C., Engstler A.J., Ziegenhardt D., Bergheim I. (2015). Supplementation of sodium butyrate protects mice from the development of non-alcoholic steatohepatitis (NASH). Br. J. Nutr..

[40] Ye J., Lv L., Wu W., Li Y., Shi D., Fang D., Guo F., Jiang H., Yan R., Ye W. (2018). Butyrate Protects Mice Against Methionine–Choline-Deficient Diet-Induced Non-alcoholic Steatohepatitis by Improving Gut Barrier Function, Attenuating Inflammation and Reducing Endotoxin Levels. Front. Microbiol..

[41] Saunder D.R. (1991). Absorption of Short-Chain Fatty Acids in Human Stomach and Rectum. Nutr. Res..

[42] Schmitt M.G., Soergel K.H., Wood C.M., Steff J.J. (1977). Absorption of Short-Chain Fatty Acids from the Human Ileum. Dig. Dis. Sci..

[43] Gill R.K., Saksena S., Alrefai W.A., Sarwar Z., Goldstein J.L., Carroll R.E., Ramaswamy K., Dudeja P.K. (2005). Expression and membrane localization of MCT isoforms along the length of the human intestine. Am. J. Physiol. -Cell Physiol..

[44] Guilloteau P., Martin L., Eeckhaut V., Ducatelle R., Zabielski R., Van Immerseel F. (2010). From the gut to the peripheral tissues: The multiple effects of butyrate. Nutr. Res. Rev..

[45] Han X., Song H., Wang Y., Sheng Y., Chen J. (2015). Sodium butyrate protects the intestinal barrier function in peritonitic mice. Int. J. Clin. Exp. Med..

[46] Wang H., Song W., Wu Q., Gao X., Li J., Tan C., Zhou H., Zhu J., He Y., Yin J. (2021). Fecal Transplantation from db/db Mice Treated with Sodium Butyrate Attenuates Ischemic Stroke Injury. Microbiol. Spectr..

[47] Bai H.-B., Yang P., Zhang H.-B., Liu Y.-L., Fang S.-X., Xu X.-Y. (2021). Short-chain fatty acid butyrate acid attenuates atherosclerotic plaque formation in apolipoprotein E-knockout mice and the underlying mechanism. Sheng Li Xue Bao.

[48] Pelgrim C.E., Franx B.A.A., Snabel J., Kleemann R., Arnoldussen I.A.C., Kiliaan A.J. (2017). Butyrate reduces HFD-induced adipocyte hypertrophy and metabolic risk factors in Obese LDLr-/-.Leiden mice. Nutrients.

[49] Ikejima K., Takei Y., Honda H., Hirose M., Yoshikawa M., Zhang Y.-J., Lang T., Fukuda T., Yamashina S., Kitamura T. (2002). Leptin receptor–mediated signaling regulates hepatic fibrogenesis and remodeling of extracellular matrix in the rat. Gastroenterology.

[50] Choi S.S., Syn W.K., Karaca G.F., Omenetti A., Moylan C.A., Witek R.P., Agboola K.M., Jung Y., Michelotti G.A., Diehl A.M. (2010). Leptin promotes the myofibroblastic phenotype in hepatic stellate cells by activating the Hedgehog pathway. J. Biol. Chem..

