# Supplementary material for: Butyrate Protects against Diet-Induced NASH and Liver Fibrosis and Suppresses Specific Non-Canonical TGF-β Signaling Pathways in Human Hepatic Stellate Cells
Source: Biomedicines. 2021 Dec 20;9(12):1954. doi: 10.3390/biomedicines9121954 (PMC8698820; doi:10.3390/biomedicines9121954)
Supplement: Supplementary file 1 [file biomedicines-09-01954-s001.zip › biomedicines-1515573-supplementary.pdf]

Supplemental data

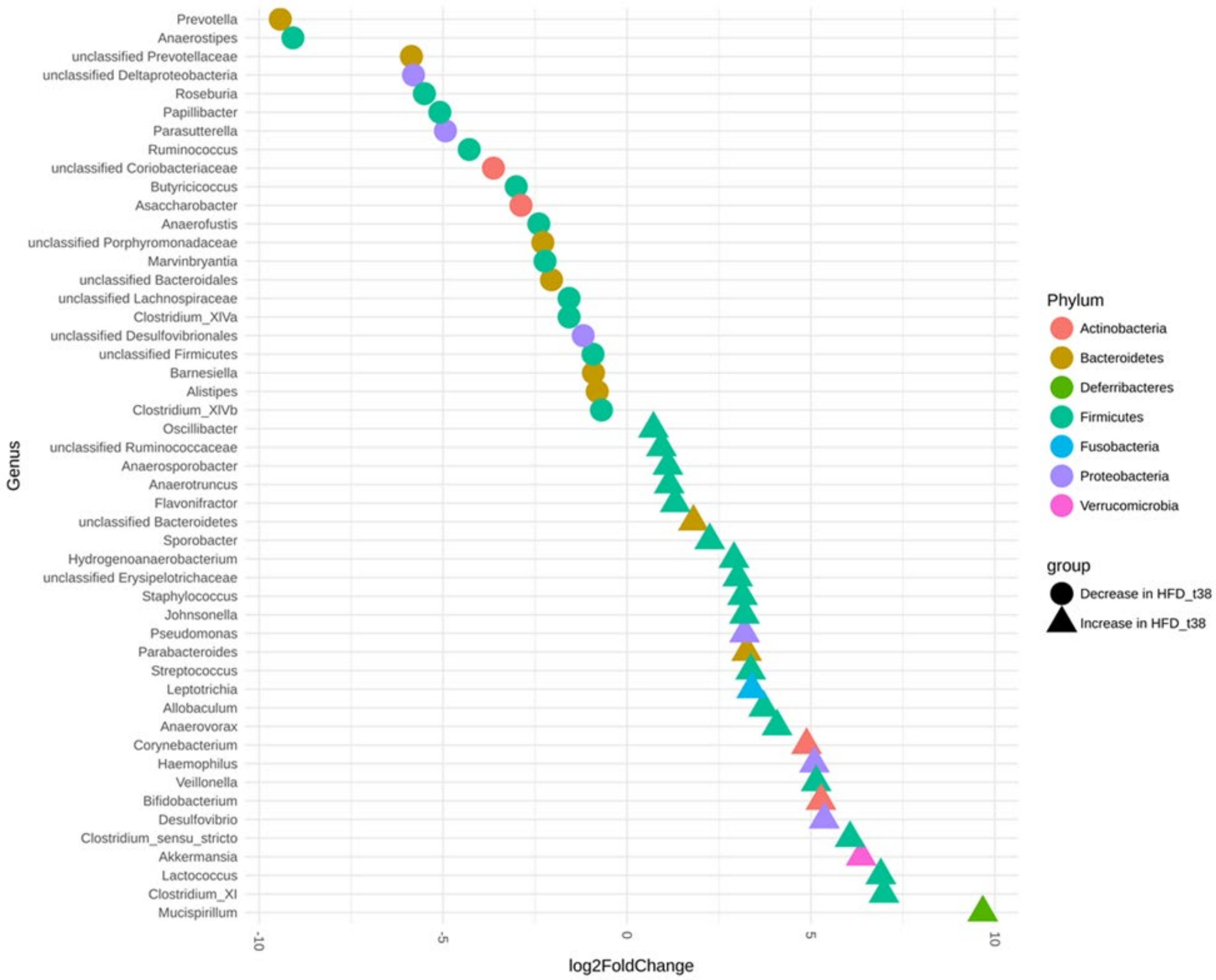

***Supplemental Figure S1: List of genera in the colon mucosa** of which the presence was significantly affected upon HFD feeding compared to chow-fed mice and expressed on a log-2fold scale.*

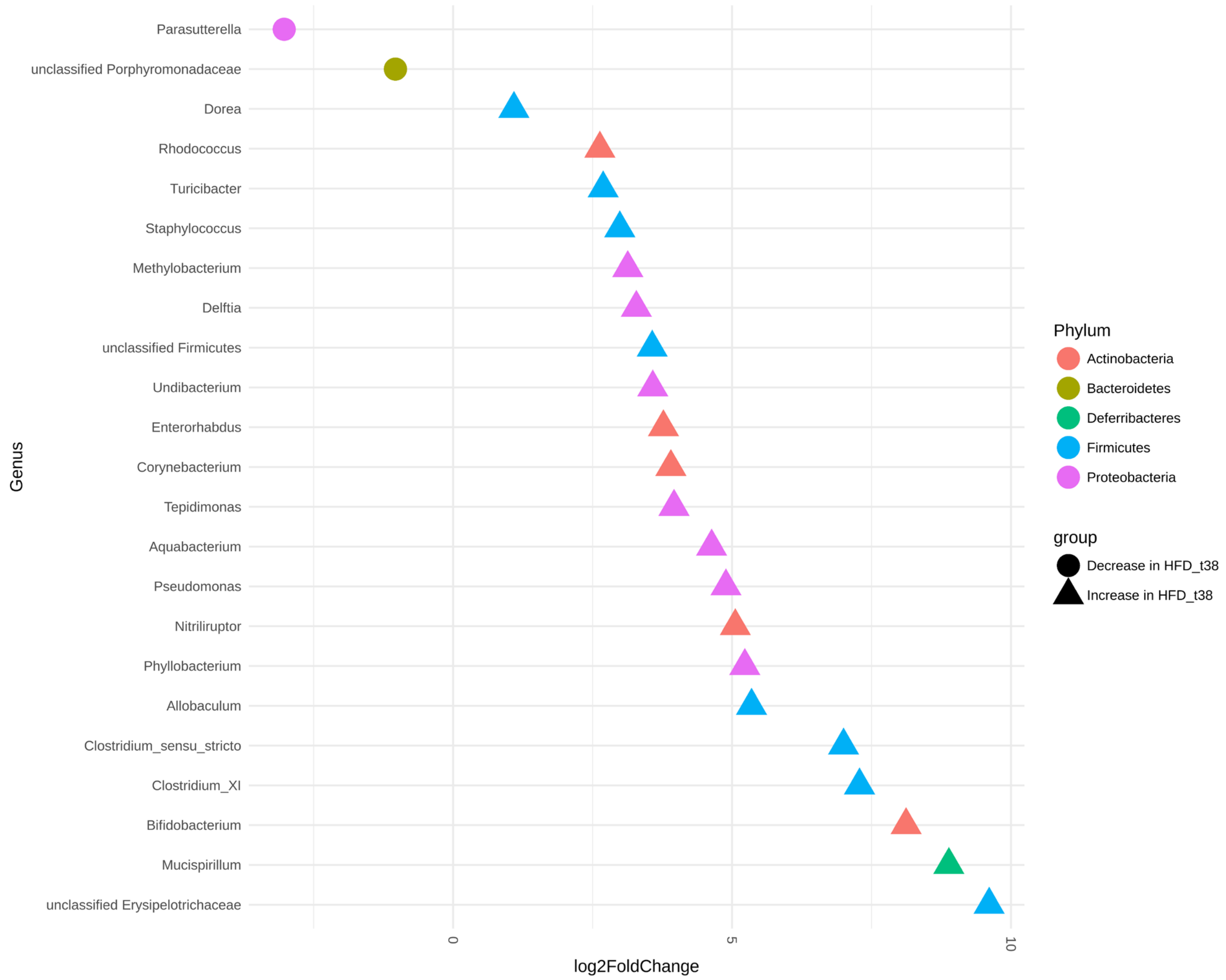

Supplemental Figure S2: Genera expressed in the mucosal layer of the ileum *of which the presence was significantly affected upon HFD feeding compared to chow-fed mice and expressed on a log2fold scale.*

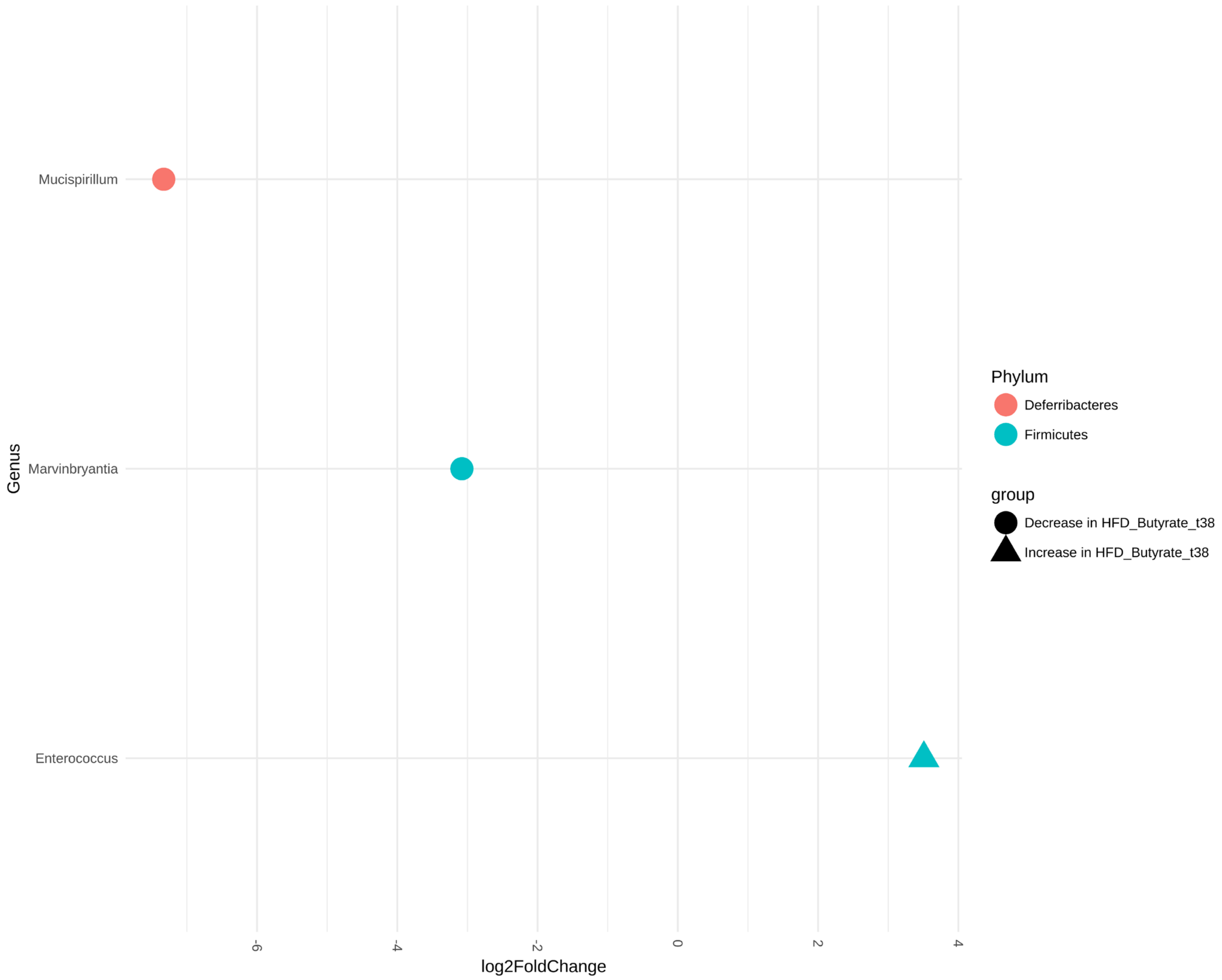

***Supplemental Figure S3: Genera expressed in the mucosal layer of the ileum** of which the presence was significantly affected upon BU feeding compared to HFD-fed mice and expressed on a log2fold scale.*

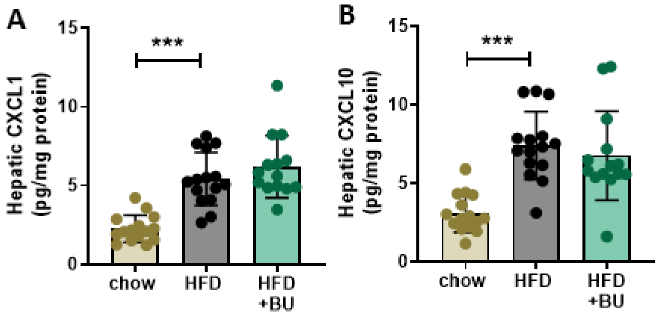

***Supplemental Figure S4: Hepatic chemokine concentrations A**) CXCL1 and **B**) CXCL10 expressed per mg protein. Data are presented as mean ± SD, *p<0.05, **p<0.01, ***p<0.001 vs the HFD control group.*

Supplementary Table S1: Circulating extracellular matrix remodeling markers

	Chow	HFD	HFD+BU
**TIMP1 (ng/ml)**	2.6 ± 1.0***	4.6 ± 1.2	3.9 ± 0.6
**PRO-C3 (ng/ml)**	11.0 ± 5.0	14.0 ± 7.2	18.5 ± 5.3
**PRO-C4 (ng/ml)**	131.7 ± 25.2*	200.5 ± 63.3	192.0 ± 50.6
**C4M (ng/ml)**	4.4 ± 1.3	5.2 ± 1.4	4.9 ± 1.0
**C6M (ng/ml)**	4.0 ± 1.5*	7.2 ± 3.3	5.8 ± 1.9

*Data are presented as mean ± SD, *p<0.05, **p<0.01, ***p<0.001 compared to the HFD-control group.*

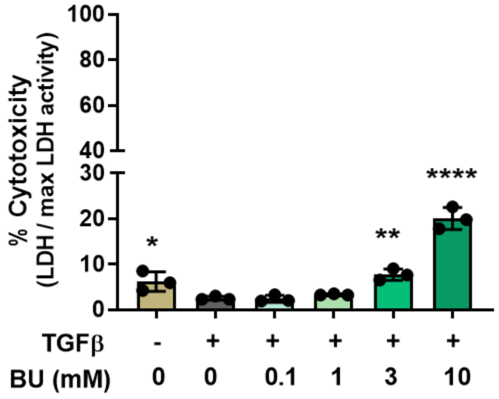

***Supplemental Figure S5: Cytotoxicity of different BU dosages** was calculated as a percentage based on maximum LDH activity in cell supernatant. Data is presented as mean with SD , *p<0.05, **p<0.01, ***p<0.001 compared to the TGF-B control group (In grey).*

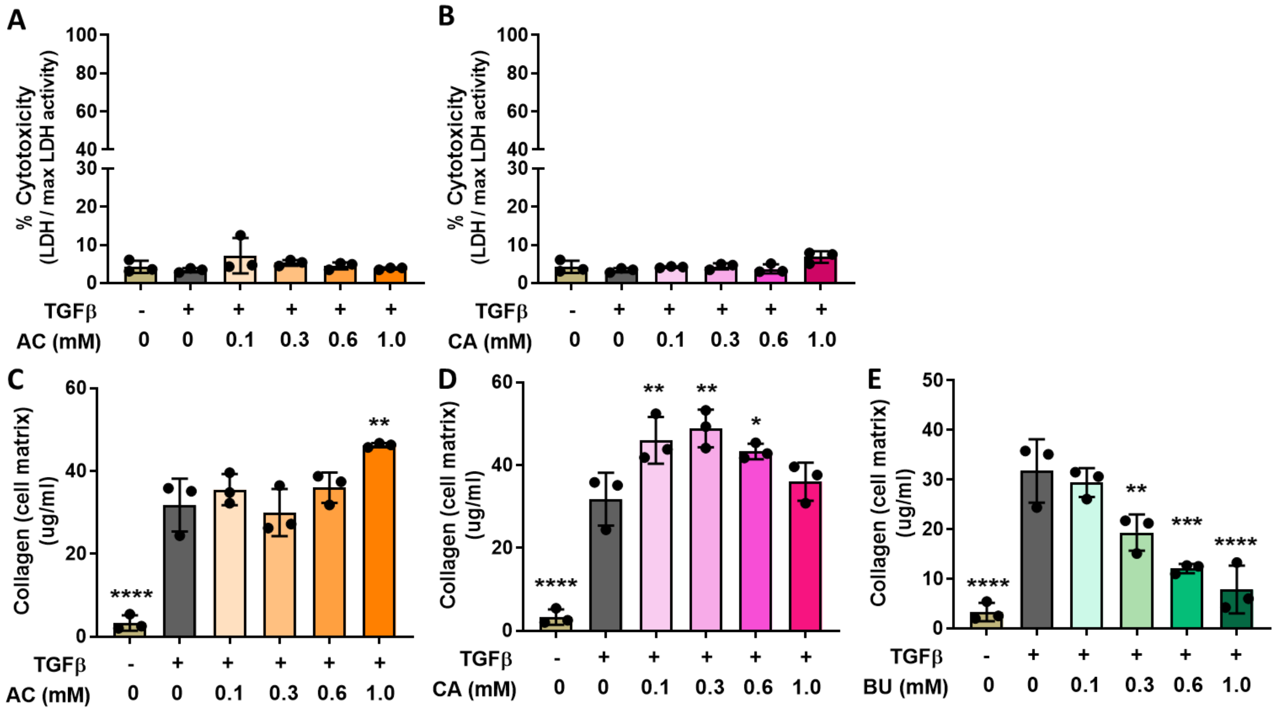

Supplemental Figure S6: Effect of short chain fatty acids acetate (AC), caproate (CA) and butyrate (BU) on TGF-β induced fibrosis in primary human hepatic stellate cells. *Cytotoxicity percentage based on maximum LDH activity in cell supernatant of cells treated with **A**) AC and **B**) CA. Also collagen content in the cell matrix was measured in cells treated with different dosages of **C**) AC, **D**) CA and **E**) BU. Data is presented as mean with SD , *p<0.05, **p<0.01, ***p<0.001 compared to the TGF-B control group (In grey).*

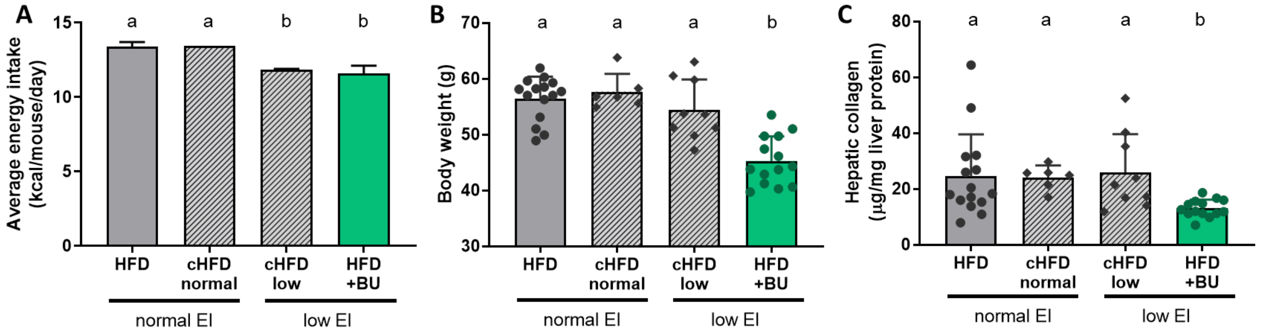

Supplemental Figure S7: Association between energy intake and liver fibrosis. *Historical data from Ldr-/-.Leiden mice fed HFD for 38 weeks (the same as in the current study) – grouped according to energy intake: cHFD normal = comparable energy intake to the HFD group in the current experiment; cHFD low = comparable energy intake to HFD+BU in the current experiment. **A**) Average energy intake, **B**) body weight and **C**) hepatic collagen content. Data are presented as mean ± SD, a indicates no significant differences, whereas b indicates a significant difference with p<0.05.*
